# Astaxanthin and its Effects in Inflammatory Responses and Inflammation-Associated Diseases: Recent Advances and Future Directions

**DOI:** 10.3390/molecules25225342

**Published:** 2020-11-16

**Authors:** Ming Xian Chang, Fan Xiong

**Affiliations:** 1 State Key Laboratory of Freshwater Ecology and Biotechnology, Key Laboratory of Aquaculture Disease Control, Institute of Hydrobiology, Chinese Academy of Sciences, Wuhan 430072, China; xiongfan@ihb.ac.cn; 2 Innovation Academy for Seed Design, Chinese Academy of Sciences, Wuhan 430072, China; 3 University of Chinese Academy of Sciences, Beijing 100049, China

**Keywords:** astaxanthin, oxidative stress, anti-inflammatory, inflammation-associated diseases

## Abstract

Astaxanthin is a natural lipid-soluble and red-orange carotenoid. Due to its strong antioxidant property, anti-inflammatory, anti-apoptotic, and immune modulation, astaxanthin has gained growing interest as a multi-target pharmacological agent against various diseases. In the current review, the anti-inflammation mechanisms of astaxanthin involved in targeting for inflammatory biomarkers and multiple signaling pathways, including PI3K/AKT, Nrf2, NF-κB, ERK1/2, JNK, p38 MAPK, and JAK-2/STAT-3, have been described. Furthermore, the applications of anti-inflammatory effects of astaxanthin in neurological diseases, diabetes, gastrointestinal diseases, hepatic and renal diseases, eye and skin disorders, are highlighted. In addition to the protective effects of astaxanthin in various chronic and acute diseases, we also summarize recent advances for the inconsistent roles of astaxanthin in infectious diseases, and give our view that the exact function of astaxanthin in response to different pathogen infection and the potential protective effects of astaxanthin in viral infectious diseases should be important research directions in the future.

## 1. Introduction

**Figure 1:**
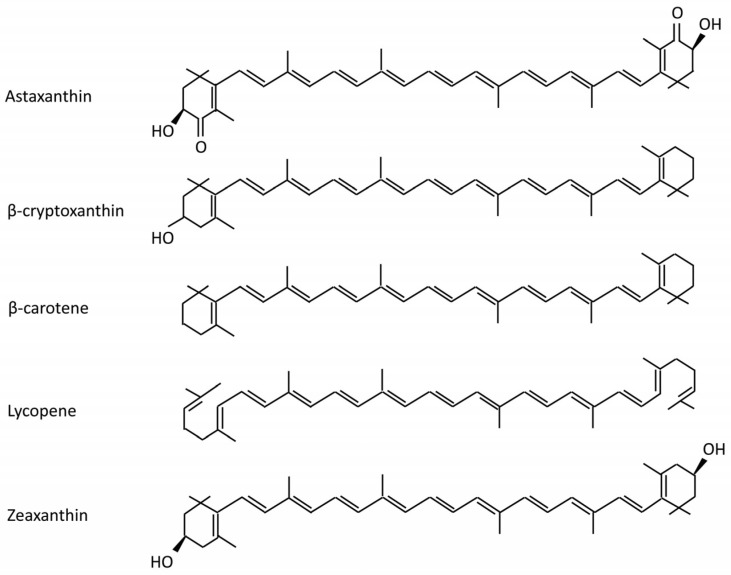
Chemical structures of selected carotenoids.

Astaxanthin is a natural lipid-soluble and red-orange oxycarotenoid pigment, and belongs to a group of carotenoids called xanthophylls, which include β-cryptoxanthin, β-carotene, lycopene, and zeaxanthin (Figure 1) [1,2]. Astaxanthin was firstly discovered in lobsters and employed in aquaculture [3]. Owing to its anti-oxidative features, astaxanthin has gained the approval as a supplement for food in 1991 [4,5]. Astaxanthin is primarily biosynthesized by microalgae, phytoplankton, yeast and bacteria [6,7], and then accumulated in zooplankton, crustaceans and subsequently fish [8,9]. Astaxanthin can be extracted in sundry seafood including algae, shrimp, krill, lobster, asteroidean, crustacean, trout, red sea bream, and salmon [10].

Astaxanthin is derived from β-carotene by 3-hydroxylation and 4-ketolation, and catalyzed by β-carotene hydroxylase and β-carotene ketolase respectively [11]. It has a molecular structure similar to that of β-carotene, with the polar ionone rings at either end of the molecule and a nonpolar zone in the middle [12]. However, in contrast to 11 in β-carotene, the possession of 13 conjugated double polyunsaturated bonds gives astaxanthin unique chemical properties, molecular structure and light absorption characteristics [10], which makes astaxanthin more polar and greatly enhances its antioxidant property [13,14]. Astaxanthin exists in three different stereoisomers, namely (3*S*, 3′*S*), (3*R*, 3′*R*) and (3*R*, 3′*S*), that differ in the configuration of two hydroxyl groups on the molecule.

Nowadays, most of available astaxanthin on the market is synthetically produced for the usages in the feeds. Synthetic astaxanthin starts from the ketoisophorone obtained from petroleum, which yields more different stereoisomers than that is naturally found [15]. Only a small part of commercial astaxanthin is extracted from *Haematococcus pluvialis* (algal production), *Xanthophyllomyces dendrorhous* (yeast production) or other astaxanthin-producing biological organisms [16]. In those organisms, the free form of astaxanthin is relatively uncommon, and most of astaxanthin is either conjugated with proteins or esterified with one or two fatty acids. In *H. pluvialis*, 99% of astaxanthin exist in the way of acyl esters, which make up nearly all the astaxanthin currently available in commercial dietary supplements [12]. However due to the early scare report, which showed that petrochemicals for astaxanthin synthesis could cause cancer, only the astaxanthin made from algal and yeast is approved for human consumption [17,18]. Synthetic forms of astaxanthin are predominantly used for animal feed.

Similar to other carotenoids, astaxanthin has numerous metabolic and physiological functions. However, astaxanthin is more bioactive than other carotenoids such as zeaxanthin, lutein, and carotene. Astaxanthin has attracted considerable interest due to its potential pharmacological effects, including a strong antioxidant property, DNA repair, stress forbearance, cell regeneration, neuroprotective, antiproliferative, anti-inflammatory, anti-apoptotic, antidiabetic, anticancer, and skin-protective effects [6,19,20]. The aim of this review is to highlight and summarize the advances toward understanding the effects of astaxanthin on inflammatory responses and inflammation-associated diseases.

## 2. The Anti-Inflammation Mechanisms of Astaxanthin

Inflammation is a biological response to harmful stimuli, such as pathogens, damaged cells, toxic compounds or irradiation, and acts by removing injurious stimuli and initiating the healing process [21]. Usually, inflammation is a defense mechanism that is vital to host health, when cellular and molecular events efficiently minimize impending injury or infection and contribute to restoration of tissue homeostasis [22]. However, nonresolving inflammation is not a primary cause but contributes to their pathogenesis for a variety of chronic diseases including chronic obstructive pulmonary disease, inflammatory bowel disease, neurodegenerative disease, atherosclerosis, or rheumatoid arthritis and so on [22]. In recent decades, a great number of inflammatory biomarkers including kinins, acute phase proteins (APPs), platelet-activating factor (PAF), prostaglandins, leukotrienes, amines, purines, cytokines, chemokines, adhesion molecules, and inflammatory signaling pathways including NF-κB, MAPK, and JAK-STAT pathways have been found [23,24].

**Figure 2:**
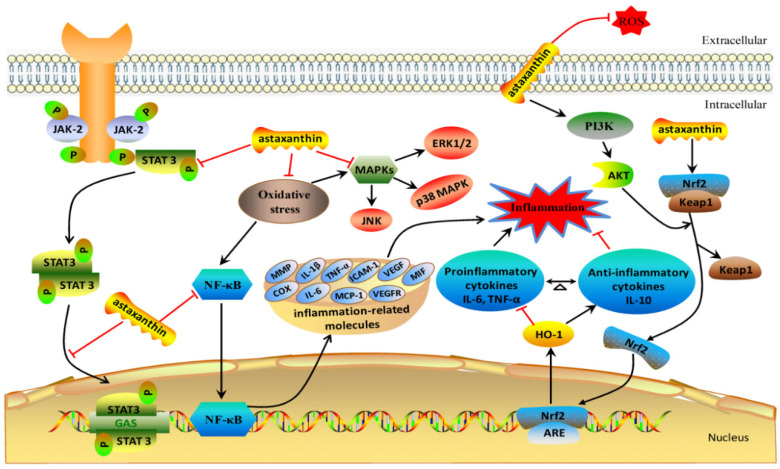
The anti-inflammation mechanisms of astaxanthin. Inflammatory biomarkers, such as many acute phase proteins (APPs), inducible enzymes, chemokines and cytokines, are target genes regulated by astaxanthin. In addition to inflammatory molecules, astaxanthin can promote PI3K/AkT and nuclear factor erythroid 2-like 2 (Nrf2) signaling pathways, but block NF-κB, extracellular-signal-regulated kinase (ERK1/2), c-Jun N-terminal kinases (JNK), p38 MAPK, and JAK-2/STAT-3 signaling pathways to attenuate inflammation. Red arrows indicate inhibitory action, and black arrows show enhancement action.

The various mechanisms of astaxanthin in the anti-inflammatory response have been demonstrated (Figure 2). Many APPs in mammals, such as C-reactive protein (CRP), lipopolysaccharide-binding protein (LBP), caeruloplasmin, haptoglobin, serum amyloid A, transferrin, collectin, fibrinogen and alpha 1-acid glycoprotein, have been used as biomarkers of inflammation and disease, which contribute to repair of tissue damage, kill infectious microbes and restore homeostasis [25,26]. Furthermore, chronic and abnormal activation of some inducible enzymes, including NADPH oxidase (NOX), inducible nitric oxide synthase (iNOS), cyclooxygenase (COX)-2, high-mobility group box 1 (HMGB1), superoxide dismutase (SOD), and glutathione peroxidase (GPx), have been shown to play vital roles in the development of some inflammatory diseases such as oncogenesis and cardiovascular disease [24,27]. In LPS-stimulated BV-2 microglial cells, astaxanthin inhibited the production of inflammatory mediators via suppressing the activation and protein degradation of iNOS and COX-2 [28]. In the streptozotocin (STZ)-induced diabetic rats, the administration of astaxanthin significantly decreased the protein expressions of COX-2, iNOS and ICAM-1, which suggested that the inhibitory effect of astaxanthin on diabetes-induced hepatic dysfunction could be derived from the inhibition of the inflammatory responses [29]. In young soccer players, astaxanthin supplementation prevented inflammation induced by rigorous physical training [30]. In human keratinocytes, astaxanthin effectively protected against UV-induced inflammation by decreasing the mRNA and protein expressions of iNOS and COX-2 [31]. All these data suggest that astaxanthin can exhibit its anti-inflammatory action by targeting for APPs and certain inducible enzymes.

Chemokines and cytokines are also target genes regulated by astaxanthin (Figure 2). The monocyte chemotactic protein 1 (MCP-1) is known to be an important chemokine for macrophage recruitment. In mouse adipose tissue, targeting for MCP-1 may prevent the perturbations associated with macrophage-induced inflammation [32]. Astaxanthin was found to suppress IκB-α degradation and NF-κB nuclear translocation, which led to significant inhibition of expression of MCP-1 and other inflammation-related molecules, including IL-6, vascular endothelial growth factors (VEGFs), intercellular adhesion molecule-1 (ICAM-1), VEGF receptor (VEGFR)-1 and VEGFR-2 [33]. Interferon-gamma (IFN-γ) is a pleiotropic cytokine involved in antiproliferative, pro-apoptotic, antitumor, autoinflammatory, and autoimmune diseases [34,35]. In ovalbumin-induced allergic asthma mice, astaxanthin treatment attenuated their airway inflammation, reduced the levels of total IgE and IgG1, and regulate the Th1/Th2 imbalance via inhibiting the release of IL-4 and IL-5 Th2 cytokines and increasing the release of IFN-γ Th1 cytokine [36]. The mRNA expression levels of IL-1β, IL-6, CCL2, and CXCL2 were significantly decreased by astaxanthin in the colonic mucosa of azoxymethane-treated mice [37]. Astaxanthin administration was found to improve the dermatitis and pruritus via the suppression of mRNA and protein expressions of eotaxin, macrophage migration inhibitory factor (MIF), IL-4, and IL-5 [38].

In addition to inflammatory biomarkers, it is most widely reported that astaxanthin can block the NF-κB-dependent signaling pathway and forestall gene expression of downstream inflammatory mediators such as IL-1β, IL-6, and tumor necrosis factor-α (TNF-α) [39,40]. Furthermore, in vivo and in vitro studies revealed that astaxanthin could influence the MAPK signaling pathway via modulating the expression and activity of extracellular-signal-regulated kinase (ERK1/2), c-Jun N-terminal kinases (JNK) and p38 MAP Kinase [41,42]. Several studies also demonstrated that the nuclear factor erythroid 2-like 2 (Nrf2) signaling plays an important role in inflammatory diseases [43,44]. In Adriamycin-induced focal segmental glomerulosclerosis (FSGS) mice, astaxanthin treatment could exert anti-inflammatory and antioxidant effects by promoting Nrf2 expression [45]. As regards in the diabetes rat model, dietary supplementation of astaxanthin improved cognitive deficits from oxidative stress, nitric oxide synthase and inflammation via promoting the expression of PI3K/AkT in the brain [46]. Astaxanthin also prevented the development and progression of hamster buccal pouch (HBP) carcinomas through the inhibition of JAK-2/STAT-3 signaling and its downstream targets cyclin D1, MMP-2, -9 and VEGF [47].

## 3. The Anti-Inflammatory Effects of Astaxanthin in Chronic and Acute Diseases

### 3.1. The Anti-Inflammatory Effects of Astaxanthin in Neurological Diseases

Alzheimer’s disease is one of the most severe chronic neurodegenerative disorders. Astaxanthin is able to act against Alzheimer’s disease. In Wistar rats with Alzheimer’s disease, astaxanthin powder from shrimp (*Litopenaeus vannamei*) shells showed a significant alleviation of cognitive functions (Table 1) [48]. In APP/PSEN1 (APP/PS1) double-transgenic mice, the administration of synthesized docosahexaenoic-acid-acylated astaxanthin diesters (AST-DHA) attenuated cognitive disorders by regulating the parameters of oxidative stress and suppressing neuroinflammation (Table 1) [49].

Parkinson’s disease (PD), which is an age-related disorder mainly caused by neuroinflammation and oxidative stress, is the second most common cause of neurodegenerative disorders. Astaxanthin was found to multi-target drug in preventing PD disease progression. In a mouse PD model from both young and aged mice, a natural compound astaxanthin was less efficacious in the aged animals, since astaxanthin failed in protecting against 1-methyl-4-phenyl-1,2,3,6-tetrahydropyridine (MPTP) neurotoxicity in aged animals (Table 1) [50]. Alpha synuclein (SNCA) is a major causative gene that responsible for the onset of PD. Astaxanthin could protect against PD-caused neuron damage by targeting miR-7/SNCA axis to suppress endoplasmic reticulum (ER) stress (Table 1) [51]. The effects of DHA-astaxanthin (DHA-acylated astaxanthin ester), non-esterified astaxanthin and DHA + astaxanthin (combination of non-esterified astaxanthin with DHA) on PD were investigated in mice with PD. The results revealed that DHA-astaxanthin significantly suppressed the PD development in MPTP-induced mice, which was better than the effects of astaxanthin and DHA + astaxanthin. Although all three astaxanthin supplements could inhibit oxidative stress, DHA-astaxanthin group had the highest inhibiting effect for the apoptosis of dopaminergic neurons through JNK and P38 MAPK pathway, which suggested that DHA-astaxanthin was superior to astaxanthin in preventing behavioral deficits via apoptosis rather than oxidative stress (Table 1) [52].

Cerebral ischemia/reperfusion (IR) can cause irreversible neuronal injuries. Several studies have investigated the effect of astaxanthin treatment in preventing the risk of ischemia on brain recovery. In a mouse model of vascular cognitive impairment (VCI), astaxanthin treatment improved learning and memory deficits after repeated cerebral IR injury, with the reduced damage of hippocampal neurons and the inhibition of neuronal apoptosis (Table 1) [53]. Preventive treatment of astaxanthin also prevented neurological deficits and reduced cerebral infarction volume through multiple mechanisms, which included the suppression of ROS (reactive oxygen species), prevention of apoptosis, activation of Nrf2–ARE defense pathway, and promotion of neural regeneration (Table 1) [54]. Compared with those in the cerebral ischemia model group (MCAO group), astaxanthin treatment can promote the axonal regeneration and improve the motor function via activating the cAMP/PKA/CREB signaling pathway (Table 1) [55]. For acute cerebral infarction (ACI) rat, the treatment with astaxanthin notably inhibited oxidative stress and increased the mRNA expression of brain-derived neurotrophic factor (BDNF) and nerve growth factor (NGF), which led to ameliorate ACI (Table 1) [56]. Astaxanthin also protected the brain from oxidative damage and reduced neuronal deficits due to cerebral ischemia reperfusion injury (IRI) (Table 1) [57]. The mechanisms of astaxanthin in neuroprotective properties against cerebral ischemia-induced apoptosis were investigated, and the in vitro data revealed that astaxanthin could confer neuroprotection against the oxygen and glucose deprivation (OGD)-induced apoptosis via the PI3K/Akt/GSK3β/Nrf2 signaling pathway (Table 1) [58].

Neuropathic pain (NP) is caused by a disease or a lesion in the somatosensory nervous system [59]. In the carrageenan-induced mice paw edema and pain, the treatment with astaxanthin from *Litopenaeus vannamei* exhibited the anti-inflammatory activities and the decreased pain (Table 1) [60]. In the in vitro and/or in vivo model of NP, astaxanthin could significantly attenuate behavioral and biochemical alterations with the decreased oxidative stress (Table 1) [61], and chronic trans-astaxanthin treatment could exert therapeutic effects on thermal hyperalgesia and co-morbid depressive-like behaviors in mice with chronic pain via its potent anti-inflammatory property (Table 1) [62]. It was also found that astaxanthin attenuated neuroinflammation and mechanical allodynia via decreasing the expression of inflammatory signaling mediators (NR2B and p-p38MAPK) and inflammatory cytokine TNF-α (Table 1) [63]. Astaxanthin was also found to decrease mechanical and thermal pain through the inhibition of ERK1/2 and the activation of AKT (Table 1) [64].

### 3.2. The Anti-Inflammatory Effects of Astaxanthin in Diabetes

Diabetes mellitus (DM) is the most common metabolic disease, and the underlying factors that lead to the development of pathologies in diabetes are involved in oxidative stress and chronic inflammation [65]. Landon et al. have reviewed the biological effects and the underlying mechanisms of astaxanthin on the prevention and the treatment of DM-associated pathologies [66]. Although the exact mechanism remains elusive, astaxanthin has been found to reduce inflammation, oxidative stress and apoptosis through the regulation of different pathways. The protective effects of astaxanthin on diabetic retinopathy (DR) and diabetic neuropathy were suggested for the inhibitory effect of astaxanthin on the inflammation through the NF-κB pathway, microvascular damages through VEGF production and apoptosis through the regulation of MAPK and PI3K/Akt pathways. The protective effects of astaxanthin on diabetic nephropathy (DN) were suggested for the inhibitory effect of astaxanthin on NF-κB translocation, transforming growth factor beta (TGF-β) production, inflammation and fibrosis. The protective effects of astaxanthin on diabetic cardiovascular complications were suggested for the inhibitory effect of astaxanthin on the inflammation through the NF-κB pathway, oxidative stress regulation through thrombosis and vasoconstriction, the levels of the oxidized low-density lipoprotein (oxLDL) and vasoconstriction [66].

### 3.3. The Anti-Inflammatory Effects of Astaxanthin in Gastrointestinal Diseases

Inflammatory bowel disease is a chronic inflammatory disease with increased risk for colorectal cancer. In the dextran sulfate sodium (DSS)-induced colitis, dietary astaxanthin significantly inhibited the occurrence of colonic mucosal ulcers and adenocarcinoma partly through inhibition of the expression of inflammatory cytokines, which included IL-1β, IL-6, COX-2 (Table 2) [67]. In C57BL/6J mice, the mRNA expression of IL-1β, IL-6, TNF-α, IL-36α, and IL-36γ, and the activation of NF-κB, AP-1, and MAPK were suppressed by dietary astaxanthin (Table 2) [68]. In C57BL/KsJ-db/db (db/db) obese mice, astaxanthin inhibited the development of colonic premalignant lesions by reducing oxidative stress, attenuating chronic inflammation, and inhibiting NF-κB activation and cell proliferation in the colonic mucosa (Table 2) [37].

### 3.4. The Anti-Inflammatory Effects of Astaxanthin in Hepatic and Renal Diseases

Several studies have reported the roles of astaxanthin in hepatic and renal diseases (Table 3). Focal segmental glomerulosclerosis (FSGS) is a specific pattern of chronic renal injury. Astaxanthin treatment exhibited significant improvements in renal functional parameters and exerted anti-inflammatory and antioxidant effects by increasing the expression of Nrf2 and inhibiting the activation of nucleotide-binding oligomerization domain-like receptor protein 3 (NLRP3) inflammasome in FSGS mouse models [45]. Astaxanthin has effects in protecting cells and/or organs from ischemia/reperfusion (IR) induced injury by the reduced oxidative stress and inflammation in kidney [69], and contrast-induced acute kidney injury (CI-AKI) by SIRT1-p53 and SIRT1/FOXO3a pathways [70,71]. In the hepatic IR injury model, astaxanthin pretreatment attenuates apoptosis and autophagy via the ROS/MAPK pathway [41], or may be involved in the inhibitory mechanism through the decrease of ROS production and inflammatory cytokine expression, and inactivation of MAPK [72]. Astaxanthin also has a protective effect in ConA-induced autoimmune hepatitis through the down-regulation of JNK/p-JNK-mediated apoptosis and autophagy [73].

### 3.5. The Anti-Inflammatory Effects of Astaxanthin in Eye and Skin Disorders

Dry eye disease (DED) has become a chronic ocular surface disease. The protective effect and potential mechanism of astaxanthin on DED were characterized, and suggested that PI3K/Akt signaling pathway may be involved in the protection of astaxanthin in dry eye via regulating the expression of HMGB1 (Table 4) [74]. Furthermore, a study showed that astaxanthin encapsulated in liposomes was effective in preventing DED through promoting antioxidative effects (Table 4) [75].

Atopic dermatitis (AD) is a common chronic inflammatory skin disease. The administration of astaxanthin can reduce the clinical skin severity score and the spontaneous scratching in AD mice via the regulation of the inflammatory effects (Table 4) [38], reduce the skin inflammation and allergic responses induced by PA treatment via inhibition of NF-κB signaling (Table 4) [76].

## 4. The Anti-Inflammatory Effects of Astaxanthin in Bacterial Infectious Diseases

Infection with *Helicobacter pylori* is a critical cause of gastrointestinal diseases, which stimulates the production of ROS and leads to the expression of inflammatory mediators in tissues [77]. The treatment of astaxanthin from shrimp cephalothorax influences cytokine release of splenocytes in *H. pylori*-infected mice with the increasing expression of IFN-γ, IL-10, and IL-2 (Table 5) [78]. Astaxanthin-rich algal meal showed an inhibitory effect on *H. pylori* growth and lower inflammation scores in a BALB/cA mouse model (Table 5) [79,80,81]. In human gastric epithelial cells, astaxanthin inhibited *H. pylori*-induced mitochondrial dysfunction and ROS-mediated IL-8 expression via activating peroxisome proliferator-activated receptors-γ (PPAR-γ) (Table 5) [82]. However, in patients with *H. pylori*, the treatment of astaxanthin had no significant effect on *H. pylori* growth or the expression of any of the interleukins, but with a significant up-regulation of CD4 and down-regulation of CD8 (Table 5) [83]. Although the inflammatory biomarkers regulated by astaxanthin are different between human and mice infected with *H. pylori*, all these data suggest the protective effects of astaxanthin against *H. pylori* infection.

## 5. Conclusions and Future Directions

Massive evidences in vivo and in vitro have showed the anti-inflammatory effects and mechanisms of astaxanthin in mammals. Astaxanthin has been confirmed to alleviate chronic and acute inflammation in various diseases, including neurodegenerative disorders, diabetes, gastrointestinal disease, renal inflammation, as well as skin and eye diseases in different experimental models, which demonstrate that astaxanthin can be an excellent candidate for treating inflammation-related diseases. Significantly, many clinical studies and reports also prove the effects of astaxanthin in cardioprotection, immune modulation, skin and cosmetic benefits, sport performance, ophthalmology and safety [84]. All these data suggest that astaxanthin will be a suitable multi-target pharmacological agent.

In addition to in mammals, astaxanthin is reported to play an important role in non-mammalian models. A dietary supplementation with astaxanthin was found to lessen immunopathology of mealworm beetle (*Tenebrio molitor*) through an immune depressive effect. The larvae fed with astaxanthin were more sensitive to the infection with *Bacillus cereus* and *B. thuringiensis* infection [85]. In common carp (*Cyprinus carpio*), dietary astaxanthin significantly increased the growth rate, respiratory burst activity, lysozyme activity and bactericidal activity. *C. carpio* fed with all astaxanthin enriched diets were more resistant for *Aeromonas hydrophila* infection [86]. The inconsistent roles of astaxanthin in infectious diseases suggest that more studies are needed to investigate the exact function of astaxanthin in response to different pathogen infection, especially in aquaculture animals.

Compared with the researches of astaxanthin in various chronic and acute diseases, only a few attempts have been made to elucidate the anti-inflammatory effects of astaxanthin in bacterial infectious diseases. There are few reports about the effect of astaxanthin in viral infection. A research showed that astaxanthin did not affect antiviral effects of IFN or ribavirin (RBV) against hepatitis C virus [87]. However, another research showed that astaxanthin has the potential antiviral effect via protecting cells from HPV L1 binding [88]. In Pacific white shrimp, astaxanthin could promote the mRNA expression of antioxidant enzyme gene and increase the resistance to white spot syndrome virus (WSSV) [89]. Significantly, since the pathogenesis and complications of many viral infectious diseases such as coronavirus disease 2019 (COVID-19) are also involved in the role of oxidative stress, inflammation, apoptosis, and autophagy [90,91,92], astaxanthin may be a promising candidate in combating viral infectious diseases. However, it’s not clear at present whether the anti-inflammatory effects of astaxanthin or other functions of astaxanthin, such as antioxidative, anti-apoptosis, and autophagy-modulatory activities, have a protective effect for viral infectious diseases, especially for COVID-19 infection. More studies are needed to investigate the potential protective effects of astaxanthin in viral infectious diseases.

## Figures and Tables

**Table 1:** The anti-inflammatory effects of astaxanthin in neurological diseases.

Model	Dosage	Biomarkers	Disease	Ref
Wistar rats	10 mg/kg body weight	Oxidative markers	Alzheimer’s Disease	[48]
APP/PS1 mice	/	Oxidative markers; inflammasome expression	Alzheimer’s Disease	[49]
Mice	Bioastin^®^ at a dose of 30 mg/kg bodyweight	MPTP neurotoxin	Parkinson’s disease	[50]
Human neuroblastoma SH-SY5Y cell line and C57BL/6 mice	5, 10, 25, and 50 μM in cell line	miR-7/SNCA axis	Parkinson’s disease	[51]
Mice with Parkinson’s disease (PD),	/	The mitochondria-mediated pathway; JNK and P38 MAPK pathway	Parkinson’s disease	[52]
Male ICR mice	10 mg/kg/day	Oxidative stress parameters; Cytochrome C, cleaved Caspase-3 and Bax	Cerebral ischemia/reperfusion (IR)	[53]
Male SD (Sprague-Dawley) rats	10 mg/kg or 5 mg/kg	Oxidative stress; antioxidant genes; assessment of cell death; cell regeneration genes	Cerebral ischemia	[54]
MCAO mice	30 mg/kg	cAMP concentration	Cerebral ischemia	[55]
Male Sprague Dawley rats	20, 40, and 80 mg/kg	Oxidative stress	Acute cerebral infarction	[56]
Adult male Sprague-Dawley rats	/	Oxidant parameter	Cerebral ischemia reperfusion injury	[57]
Human SH-SY5Y cells	5, 10, 20 and 40 μmol/L	PI3K/Akt/GSK3β/Nrf2 signaling	Cerebral ischemia	[58]
Male ICR mice	50, 100, 150 mg/kg	ROS	Edema and pain	[60]
Rat C6 glial cells; Adult male Sprague Dawley rats	5 and 10 mg/kg	ROS	Neuropathic pain	[61]
Chronic constriction injury (CCI) mice	80 mg/kg	IL-1β, IL-6 and TNF-α	Neuropathic pain	[62]
Adult male Wistar rats	10 μL of 0.2 mM	NR2B, p-p38MAPK and TNF-α	Neuropathic pain	[63]
Spinal cord injury (SCI) rats	/	ERK1/2, AKT	Neuropathic pain	[64]

**Table 2:** The anti-inflammatory effects of astaxanthin in gastrointestinal diseases.

Model	Dosage	Biomarkers	Disease	Ref
Male ICR mice	50, 100, 200 ppm in diet	NF-κB, IL-1β, IL-6, COX-2	dextran sulfate sodium (DSS)-induced colitis	[67]
C57BL/6J mice	0.02 or 0.04% in diet	IL-1β, IL-6, TNF-α, IL-36α, IL-36γ, NF-κB, AP-1, ERK1/2, p38 MAPK, JNK	dextran sulfate sodium (DSS)-induced colitis	[68]
C57BL/KsJ-db/db obese mice	200 ppm in diet	IL-1β, IL-6, CCL2, CXCL2, NF-κB	azoxymethane-induced colonic premalignant lesions	[37]

**Table 3:** The anti-inflammatory effects of astaxanthin in hepatic and renal diseases.

Model	Dosage	Biomarkers	Disease	Ref
Male Balb/c mice	50 mg/kg	Nrf2, NLRP3, IL-1β, IL-18	Adriamycin-induced FSGS	[45]
Male ICR mice	5 mg/kg/day	TNF-α, IL-1β, IL-6	Ischemia/reperfusion (IR) induced injury	[69]
Male Sprague Dawley (SD) rats	/	Oxidative stress indicators, antioxidant stress indicators	Contrast-induced acute kidney injury (CI-AKI)	[70]
Male Sprague Dawley rats	50 and 100 mg/kg	Oxidative stress markers and apoptosis-related proteins	Contrast-induced acute kidney injury (CI-AKI)	[71]
Male Balb/C mice	30 mg/kg or 60 mg/kg	ROS, inflammatory cytokines and MAPK proteins	Hepatic ischemia reperfusion (IR)	[41]
Male C57BL/6 mice	25 mg/kg	ROS, inflammatory cytokines, MAPK and apoptosis-related proteins	Hepatic ischemia reperfusion (IR)	[72]
Male Balb/c mice	20 mg/kg and 40 mg/kg	NF-κB p65, TNF-α, IL-6, IL-1β, IFN-γ, autophagy and apoptotic proteins	ConA-induced autoimmune hepatitis	[73]

**Table 4:** The anti-inflammatory effects of astaxanthin in skin and eye disorders.

Model	Dosage	Biomarkers	Disease	Ref
BALB/c mice	1-μL drop of 5 μM	HMGB1, TNF-α, IL-1β, PI3K/Akt	Dry eye disease	[74]
Male Sprague-Dawley rats	200 µM	DED-related factors	Dry eye disease	[75]
Male NC/Nga mice	100 mg/kg	Eotaxin, MIF, IL-4, IL-5 and L-histidine decarboxylase	Atopic dermatitis	[38]
HR-1 mice	10 μg or 20 μg/cm^2^	IL-1β, IL-6, TNF-α, IgE, COX-2, NF-κB, iNOS	Atopic dermatitis	[76]

**Table 5:** The anti-inflammatory effects of astaxanthin in infectious diseases.

Model	Dosage	Biomarkers	Disease	Ref
BALB/c female mice	10 or 40 mg/d	IFN-γ, IL-2 and IL-10	*Helicobacter pylori* infection	[78]
BALB/cA mice	10, 50, and 100 mg/kg	Bacterial load, the numbers of inflammatory cells	*Helicobacter pylori* infection	[79]
Balb/cA mice	200 mg per kg body weight per day	IFN-γ, IL-4, IL-2, bacterial load	*Helicobacter pylori* infection	[80]
Female BALB/c mice	100 mg/kg	IFN-γ, IL-4, bacterial load	*Helicobacter pylori* infection	[81]
Human gastric epithelial cell line AGS	5 µM	ROS, NF-κB, IL-8, PPAR-γ	*Helicobacter pylori* infection	[82]
Patients	40 mg daily	CD4, CD8	*Helicobacter pylori* infected	[83]
